# Wingless-type family member 3A triggers neuronal polarization via cross-activation of the insulin-like growth factor-1 receptor pathway

**DOI:** 10.3389/fncel.2013.00194

**Published:** 2013-10-25

**Authors:** María E. Bernis, Mariana Oksdath, Sebastián Dupraz, Alvaro Nieto Guil, Marisa M. Fernández, Emilio L. Malchiodi, Silvana B. Rosso, Santiago Quiroga

**Affiliations:** ^1^Departamento de Química Biológica-CIQUIBIC, Fac. de Ciencias Químicas, Universidad Nacional de Córdoba-CONICET, Córdoba, Argentina; ^2^Cátedra de Inmunología and Instituto de Estudios de la Inmunidad Humoral (IDEHU), CONICET-UBA, Facultad de Farmacia y Bioquímica, UBA, Buenos Aires, Argentina

**Keywords:** Wnt3a, IGF-1 receptor pathway, Frizzled-7, axonal outgrowth, neuronal polarity

## Abstract

Initial axonal elongation is essential for neuronal polarization and requires polarized activation of IGF-1 receptors (IGF-1r) and the phosphatidylinositol 3 kinase (PI3k) pathway. Wingless-type family growth factors (Wnts) have also been implied in the regulation of axonal development. It is not known, however, if Wnts have any participation in the regulation of initial axonal outgrowth and the establishment of neuronal polarity. We used cultured hippocampal neurons and growth cone particles (GCPs) isolated from fetal rat brain to show that stimulation with the wingless family factor 3A (Wnt3a) was sufficient to promote neuronal polarization in the absence of IGF-1 or high insulin. We also show that Wnt3a triggered a strong activation of IGF-1r, PI3k, and Akt in developmental Stage 2 neurons and that the presence of activatable IGF-1r and PI3k activation were necessary for Wnt3a polarizing effects. Surface plasmon resonance (SPR) experiments show that Wnt3a did not bind specifically to the IGF-1r. Using crosslinking and immuno-precipitation experiments, we show that stimulation with Wnt3a triggered the formation of a complex including IGF-1r-Wnt3a-Frizzled-7. We conclude that Wnt3a triggers polarization of neurons via cross-activation of the IGF-1r/PI3k pathway upon binding to Fz7.

## Introduction

The establishment of neuronal polarity requires the action of two interrelated processes: axon specification and elongation. The initial signals and pathways that determine polarity are beginning to be understood. A particularly early event that occurs in neurons that have not yet exhibited a discernible axon (stage 2 of differentiation) is the segregation of activatable, membrane inserted IGF-1 receptors (IGF-1r) in a single neurite (Sosa et al., 2006). Subsequently, phosphatidylinositol-3 kinase (PI3k) and its product, phosphatidylinositol 3,4,5-trisphosphate (PIP3), accumulate in the distal region and growth cone of the neurite with the IGF-1r. These events are critical for the outgrowth of the future axon, together with activation of PI3k by IGF-1r (Shi et al., 2003; Sosa et al., 2006). Additionally, it has been shown that IGF-1 produces a robust and long lasting activation of the PI3k-Akt pathway through activation of the IGF-1r in hippocampal neurons (Zheng and Quirion, 2004). Activation of IGF-1r by their cognate ligand (IGF-1) and by insulin is also able to transactivate other receptor systems, such as epidermal growth factor receptors (EGFRs) (Roudabush et al., 2000).

Growth factors belonging to the Wnt family have been implicated in different aspects of axonal development (Rosso and Salinas, 2007). There is increasing published evidence indicating that Wnts are key mediators of axonal outgrowth and guidance (Lyuksyutova et al., 2003; Arevalo and Chao, 2005) and regulate axonal remodeling through inhibition of GSK3β (Krylova et al., 2000; Ciani et al., 2004). Interestingly one study showed that Wnt5a could accelerate initial axonal outgrowth by activating aPKC and stimulating the interaction between Dv1 and the polarity complex PAR3/PAR6/aPKC (Zhang et al., 2007). However, the above mentioned experiments were made in the presence of high insulin concentration, enough to fully activate the IGF-1r and trigger hippocampal neuron polarization in the absence of any other growth factor (Dotti et al., 1988; Sosa et al., 2006). Another member of the Wnt family, Wnt3a, has been shown to be essential for hippocampal development (Lee et al., 2000) and regulates neurite outgrowth in neuroblastoma cells (Greer and Rubin, 2011). The experiments in the present paper were designed to investigate the involvement of Wnt family members (specifically Wnt3a) in the regulation of initial axonal outgrowth and the establishment of neuronal polarity. Moreover, we studied a possible relationship between Wnt3a and the IGF-1r pathway.

Using cultured hippocampal pyramidal neurons and growth cone particles (GCPs) prepared from fetal rat brain (GCPs), our results show that Wnt3a strongly stimulated the IGF-1r and the PI3k pathway through the formation of an IGF-1r (active)-Wnt3a-Frizzled-7 (Fz7) complex and, therefore, stimulated initial axonal outgrowth and the establishment of neuronal polarity.

## Materials and methods

### Primary antibodies

The following primary antibodies were used: affinity-purified rabbit polyclonal antibody against βgc (Quiroga et al., 1995), which was diluted 1:200 for immunofluorescence and 1:500 for western blotting; mouse monoclonal antibody against IGF-1r clone αIR3 (Calbiochem), which was diluted 1 μg/ml for immunoprecipitation; rabbit monoclonal antibody against Frizzled-7 receptor (Sigma-Aldrich), which was diluted 1:500 for western blotting; rabbit polyclonal antibody to the phosphorylated (tyr 458) binding motif of p85 PI3k (Cell Signaling), which was diluted 1:200 for immunofluorescence and 1:1000 for western blotting; anti PI3k p85 rabbit monoclonal antiserum (Cell Signaling), which was diluted 1:1000 for western blotting; mouse monoclonal antibody against TrkB (Santa Cruz Biotechnology), which was diluted 1:100 for western blotting; rabbit polyclonal antibody to phosphorylated IGF-1r (Tyr 980) (Cell Signaling), which was diluted 1:100 for immunofluorescence and 1:1000 for western blotting; goat monoclonal antibody against phosphorylated TrkB receptor (Santa Cruz Biotechnology), which was diluted 1:200 for western blotting; rabbit polyclonal antibody against the Ror2 receptor (Cell Signaling), which was diluted 1:500 for western blotting; rabbit monoclonal antibody against Wnt3a (Cell Signaling), which was diluted 1:50 for immunoprecipitation and 1:500 for western blotting; mouse monoclonal antibody against GAP-43 (Sigma-Aldrich), which was diluted 1:500 for western blotting; mouse monoclonal antibody to the axonal marker Tau-1 (Calbiochem), which was diluted 1:800 for immunofluorescence; rabbit monoclonal antibody to β-III-tubulin (Sigma-Aldrich), which was diluted 1:6000 for immunofluorescence and 1:4000 for western blotting; mouse monoclonal antibody (clone 9F10) to c-myc (Roche Diagnostics, Germany) which was diluted 1:400 for immunofluorescence; rat monoclonal antibody to neural cell adhesion molecule L1 clone 324 (Millipore) 1 μg/ml and rabbit polyclonal antibody to phosphorylated Akt (Santa Cruz Biotechnology), which was diluted 1:500 for western blotting.

### Animals

All animal procedures were performed following approved protocols by the Board of Animal Welfare, School of Chemical Sciences, National University of Córdoba, Argentina.

### Cell culture

Dissociated hippocampal pyramidal cells were prepared from embryonic rat brain and cultured as previously described (Mascotti et al., 1997). To allow for neuronal survival, the medium contained a low level of insulin (5 nM), which was sufficient to stimulate insulin receptors but not fully activate IGF-1r [It has been reported that the affinity of IGF-1r for insulin is 100–500-fold lower than for its cognate ligand IGF-1 (Ballard et al., 1988); previous published experiments from our laboratory indicated that insulin significantly induced neuronal polarization at concentrations of 100 nM or higher (Grasso et al., 2013)]. Where indicated, 1.35 nM Wnt3a (Purro et al., 2008) or 20 nM IGF-1 [we have previously shown that IGF-1r was fully activated by IGF-1 at concentrations of 10 nM or higher (Quiroga et al., 1995)] was added to the culture medium. Neurons were considered to be at stage 3 when the length of the axon (positive to immunostaining with Tau-1) exceeded that of the average length of minor neurites by at least 20 μm (Craig and Banker, 1994).

### Cell transfection

cDNAs encoding shRNAs were inserted in a dicistronic vector pSuper.neo + GFP (pSuper RNAi System-OligoEngine) under the control of the H1 RNAIII polymerase promoter, and the transfection marker GFP was under the control of the PGK promoter. The target DNA sequence was GCCCATGTGTGAGAAGACC (Bohula et al., 2003; Sosa et al., 2006). A scrambled DNA target sequence (GAACGGTCGCAGTGTACCA) was created using the siRNA WizardTM, InvivoGen. The resulting plasmids were referred to as IGF-1r shRNA and scrambled sequence RNA (ssRNA). The Fz7 CRD was a generous gift from Dr. P. Salinas. The myc tagged, constitutively active p110 construct was a generous gift from Dr. L. Williams. The plasmids were mixed with Lipofectamine 2000 and added to the neurons 2 h after plating.

### Blocking IGF-1r with antibodies

Neurons were grown in the presence of the mouse monoclonal antibody clone αIR3 (diluted to 1 μg/ml), which blocks the activation of IGF-1r. Clone αIR3 binds to an epitope within the α-subunit adjacent to the ligand binding site and blocks IGF-1 from binding to its receptor without affecting either insulin or IGF-II receptors (Kull et al., 1983; Rohlik et al., 1987). Clone αIR3 cross-reacts with the α-subunit of rat IGF-1r and has been extensively used in IGF-1r blocking experiments (Linseman et al., 2002; Sosa et al., 2006). Fresh antibody was added to the culture medium every 8 h.

### PI3k inhibition

Neurons were grown in the presence (20 μ M) or absence of the specific pharmacologic inhibitor of PI3k enzyme LY294002 (Sigma-Aldrich).

### Immunofluorescence

Cells were fixed and processed as previously described (Dupraz et al., 2009). Cells were observed using a Zeiss Pascal 5 confocal microscope. Images were captured and digitized using LSM Image software, all images were printed using Adobe Photoshop. The images were analyzed using ImageJ and/or StatSoft software.

### Immunofluorescence of active IGF-1r and active PI3k

Cells were cultured as previously described. After 12 h in culture, cells were stimulated for 5 min with 20 nM IGF-1 or 1.35 nM Wnt3a, fixed, and processed for immunofluorescence using an antibody selective for the phosphorylated form of IGF-1r (Sosa et al., 2006) or a specific antibody against the phosphorylated form of the PI3k regulatory subunit p85 (Sosa et al., 2006).

### Isolation of growth cones

Axonal growth cones were isolated from developing brain as previously described (Pfenninger et al., 1983; Lohse et al., 1996). Briefly, brains of fetal rats at 18 days of gestation were homogenized. A low-speed supernatant (LSS) was prepared, loaded onto a discontinuous sucrose density gradient with steps of 0.83 M and 2.66 M sucrose, and spun to equilibrium at 242,000 g. The fraction at the load/0.83 M interface (designated “A”) contained the GCPs.

### Immunoprecipitation experiments

Intact GCPs were incubated for 30 min in the presence or absence of Wnt3a (1.35 nM). To stabilize the ligand-receptor complexes, we used the membrane insoluble and cleavable crosslinker DTSSP, Pierce Protein Research Product, (1 mM) following the manufacturer protocols. GCPs were spun down for 6 min at 4000 g, and the pellet was lysed with 1X RIPA buffer containing 3 mM vanadate and a protease inhibitor cocktail. For immunoprecipitation, the supernatant was incubated with the indicated antibodies for 2 h at 4°C before adding protein A/G plus-coated beads. In some experiments (Figure 7D), total protein from hippocampal neurons in culture (in control conditions or stimulated with Wnt3a) was used as starting material.

### Gel electrophoresis and western blot analysis

Proteins were separated by SDS-polyacrylamide gel electrophoresis, transferred to PVDF membranes and developed with ECL on x-ray films as previously described (Dupraz et al., 2009), or were analyzed using a Li-COR Odyssey Infrared Imaging System (LI-COR Biosciences) according to the manufacturer's instruction.

### Surface plasmon resonance (SPR) assay

The interaction of soluble recombinant human Insulin-like Growth Factor-1 (IGF-1) with its receptor (IGF-1r-recombinant human Met 1-Asn 932 extracellular moiety; R&D System, Minneapolis, USA) was measured by SPR analysis using a Biacore T100 instrument (Biacore Inc, Piscataway, NJ), that allows to determine the interactions between two molecules in real time (Karlsson et al., 1991). IGF-1r (ligand, 1.0 μg/ml) was dialyzed against 10 mM sodium acetate pH 4.5 and coupled to the carboxymethyl-dextran matrix of CM5 sensor chips (Biacore) using the Amine Coupling Kit as described (Johnsson et al., 1991). The activation and immobilization periods were set between 3 and 7 min to couple the desired amount of proteins yielding between 1200 and 2000 resonance units (RU). Recombinant human IGF-1 (analyte) was dialyzed against phosphate-buffered saline (PBS) containing 150 mM NaCl and twofold dilutions were made in the same buffer.

In order to characterize the direct interaction between IGF-1r and mouse Wnt3a or Fz7 [Recombinant Human FZD7/frizzled-7 Protein, made in HEK293 (aa 45–169-Speed Biosystems, MD, USA) preincubated with Wnt3a] IGF-1r was immobilized on a CM5 sensor chip surface, as described above. Recombinant Wnt3a was reconstituted in PBS-0.55% of bovine serum albumin (BSA), and twofold dilutions were made in the same buffer.

All binding experiments were performed at 25°C. Dissociation was carried out in PBS. Pulses of PBS were used to regenerate the surface. SPR data were analyzed using Biacore T100-evaluation 2.0.1 software (Biacore). All the experiments were repeated at least three times and the standard deviations were typically less than 10%. Dissociation constants (K_D_) and association and dissociation rates (*kon* and *koff*, respectively), were determined under kinetic conditions after correction for non-specific binding, in which the proteins were passed over blocked, no immobilized surfaces, as previously described (Fernandez et al., 2007, 2011).

## Results

### Wnt3a triggered polarization of hippocampal neurons in culture as well as polarized activation of IGF-1r and PI3k

**Figure 1:**
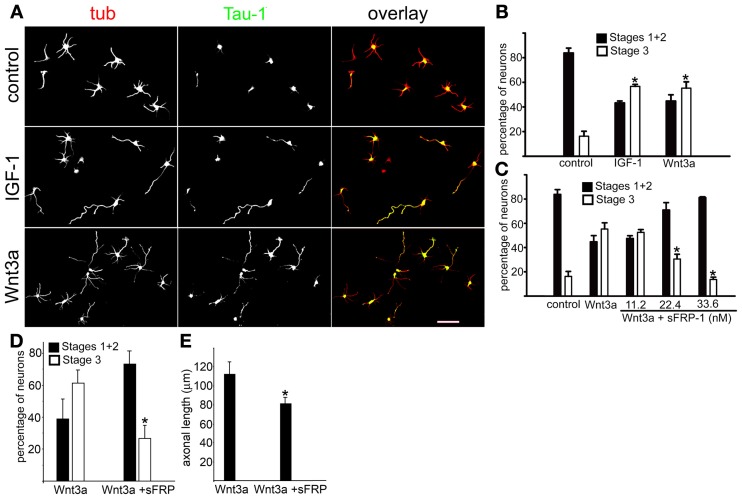
**Wnt3a triggered hippocampal neuron polarization. (A)** Double immunofluorescence micrographs of hippocampal neurons after 20 h in culture showing the distribution of the neuronal marker β-III tubulin (tub) and the axonal marker Tau-1. Cells were cultured in control medium (5 nM insulin, top) or in medium containing either 20 nM IGF-1 (middle) or 1.35 nM Wnt3a (bottom). **(B)** Percentage (±sem) of neurons at different stages of differentiation grown for 20 h in control medium or in the presence of either 20 nM IGF-1 or 1.35 nM Wnt3a (*n* = 3 independent experiments). At least 100 cells were scored for each condition. ^*^p < 0.005 compared to control. **(C)** Percentage (±sem) of neurons at different stages of differentiation grown in control medium or in the presence of 1.35 nM Wnt3a or 1.35 nM Wnt3a plus different concentrations of sFRP-1. Note the significant dose-dependent inhibition of Wnt3a-mediated polarizing effects of sFRP-1 (*n* = 3 independent experiments). At least 100 neurons were scored for each condition. ^*^p < 0.005 compared to control. Scale bar 100 μm. **(D)** Percentage (±sem) of neurons at different stages of differentiation grown for 20 h in the presence of 1.35 nM Wnt3a and for further 24 h in the presence (or not) of 33.6 nM sFRP-1 (*n* = 3 independent experiments) At least 100 neurons were scored for each condition. ^*^p < 0.02 compared to Wnt3a. Neurons were considered to be at Stage 3 when the length of one of the processes positive to Tau-1 exceeded that of the average minor neurite by at least 20 μm. **(E)** Average length (±sem) of axons from neurons treated as in panel **(D)**. (*n* = 3 independent experiments) At least 100 neurons were scored for each condition. ^*^p < 0.02 compared to Wnt3a.

IGF-1r (activated by IGF-1 and/or insulin) controls initial axonal elongation in hippocampal neurons by activating the PI3k/Akt/Cdc42 pathway (Sosa et al., 2006). To study the possible involvement of the Wnt growth factor family Wnt3a on this process, we cultured pyramidal hippocampal neurons in “control” conditions (in the presence of 5 nM insulin), and in the presence of either 20 nM IGF-1 or 1.35 nM Wnt3a. Figure 1A shows that most neurons cultured in control conditions for 20 h failed to form axons; only short, minor neurites were present (Figure 1A, top). In contrast, most cells cultured in the presence of IGF-1 (Figure 1A, middle) or Wnt3a (Figure 1A, bottom) generated long, axon-like processes that were enriched in Tau-1 protein. To analyze this observation quantitatively, we scored the differentiation stages of neurons cultured for 20 h in control conditions or in the presence of IGF-1 or Wnt3a. We found that over 80% of the cells cultured in control conditions remained in stages 1 or 2 of differentiation, and less than 20% had formed a discernible axon (stage 3). In contrast, over 55% of the cells cultured in either the presence of IGF-1 or Wnt3a showed an identifiable Tau-1 containing axon (Figure 1B). To determine the specificity of Wnt3a axogenic effects, we used the Wnt pathway antagonist secreted frizzled-related protein 1 (sFRP-1), which binds to Wnt proteins (Rattner et al., 1997). Our results showed that sFRP-1 dose-dependently inhibited Wnt3a polarizing effects (Figure 1C). To investigate the possible role of Wnt3a in axon maintenance, we scored the differentiation stages in neurons cultured for 20 h in the presence of 1.35 nM Wnt3a (at this time over 50% of the neurons were in stage 3-see Figure 1B). Then, 33.6 nM sFRP1 was added to half of the cultures and the neurons were grown for further 24 h. We found that over 60% of the neurons cultured in the presence of Wnt3a for 44 h were in stage 3 of differentiation, in contrast less that 30% of the cells were in this stage when sFRP-1 was added to the cultures (Figure 1D). We also measured axonal length in neurons cultured as in Figure 1D. Our results indicated a significant reduction of axonal outgrowth in the neurons treated with sFRP-1 (Figure 1E).

**Figure 2:**
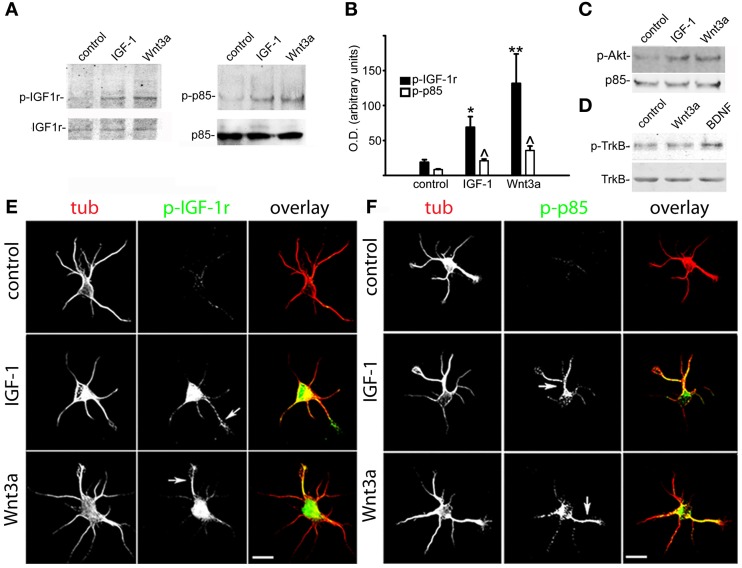
**Wnt3a elicited the polarized activation of IGF-1r and PI3k in hippocampal neurons. (A)** Western blots of active (phosphorylated) IGF-1r (left) and phosphorylated p85 (right) in control GCPs and GCPs stimulated with either 20 nM IGF-1 or 1.35 nM Wnt3a. Growth cone particles were incubated for 30 min on ice with growth factors and incubated at 37°C for 5 min in the presence of 1 mM ATP. Note the noticeable increment in both the active form of IGF-1r (left) and phosphorylated p85 (right). IGF-1r (left) or total p85 (right) were included as loading controls. **(B)** Relative optical densities of active IGF-1r and phosphorylated p85 in the western blots [similar to those shown in panel **(A)**]. Note the significant increment of both active IGF-1r and phosphorylated p85 in the GCPs stimulated with IGF-1 (^*^p < 0.01; ^∧^p < 0.05) or Wnt3a (^**^p < 0.001; ^∧^p < 0.05) compared with control. Values are the average + sem of three independent experiments. **(C)** Western blots of active (phosphorylated) Akt in control GCPs or GCPs stimulated with either 20 nM IGF-1 or 1.35 nM Wnt3a. Growth cone particles were kept on ice for 30 min with the growth factors and incubated at 37°C for 5 min in the presence of 1 mM ATP, p85 was included as a loading control. **(D)** Western blots of active (phosphorylated) TrkB receptors in control GCPs or GCPs stimulated with 20 nM IGF-1, 1.35 nM Wnt3a, or 50 ng/ml BDNF. Growth cone particles were kept on ice for 30 min with the growth factors and incubated at 37°C for 5 min in the presence of 1 mM ATP. Total TrkB was included for comparison. **(E)** Double immunofluorescence micrographs of hippocampal neurons after 12 h in culture showing the distribution of the neuronal marker β-III tubulin (tub) and active IGF-1r (p-IGF-1r). Cells were cultured in control medium (control) and challenged for 5 min with either 20 nM IGF-1 (middle) or 1.35 nM Wnt3a (bottom). Note that active IGF-1r was polarized to one neurite in the cells challenged with either IGF-1 (middle arrowhead) or Wnt3a (bottom arrowhead) in stage 2 neurons that had not exhibited a discernible axon. **(F)** Double immunofluorescence micrographs of hippocampal neurons after 12 h in culture showing the distribution of the neuronal marker β-III tubulin (tub) and phosphorylated p85 (p-p85). Cells were cultured in control medium (control) and challenged for 5 min with 20 nM IGF-1 (middle) or 1.35 nM Wnt3a (bottom). Note that phosphorylated p85 was polarized to one neurite in the cells challenged with either IGF-1 (middle arrowhead) or Wnt3a (bottom arrowhead) in stage 2 neurons that had not exhibited a discernible axon. Scale bars: 20 μm.

Next, we investigated the possible relationship between Wnt3a axogenic effects and the activation of the IGF-1r/PI3k pathway. We initially analyzed the activation of IGF-1r in GCPs challenged with 20 nM IGF-1 or 1.35 nM Wnt3a using a specific antibody for the active (phosphorylated) form of the IGF-1r. We found that Wnt3a significantly activated IGF-1r to a higher degree than the cognate ligand IGF-1 (Figure 2A, left). In addition, Wnt3a also significantly activated PI3k (Figure 2A, right). Quantification of these results (by measuring the optical density of the different bands in the western blots) is shown in Figure 2B. Our results also showed a noticeable activation of Akt, a kinase downstream of PI3k (Figure 1C). We also analyzed the possible Wnt3a-induced activation of another tyrosine-kinase receptor, TrkB (the BDNF receptor), which is enriched at the growth cone of neurons (Pfenninger et al., 2003). Our results show that Wnt3a did not cause any noticeable activation of TrkB (Figure 2D). In the present study, we found that challenging the neurons with 1.35 nM Wnt3a resulted in a distribution of active IGF-1r that was essentially identical to that achieved upon challenge with 20 nM IGF-1 (Figure 2E-middle and bottom). We also found that challenging with either 20 nM IGF-1 or 1.35 nM Wnt3a resulted in a polarized distribution of phosphorylated p85, the PI3k regulatory subunit, to one neurite of cells in stage 2 (Figure 2F).

### Triggering of initial axonal outgrowth and the establishment of neuronal polarity by wnt3a required the presence of activatable IGF-1r and activation of PI3k

**Figure 3:**
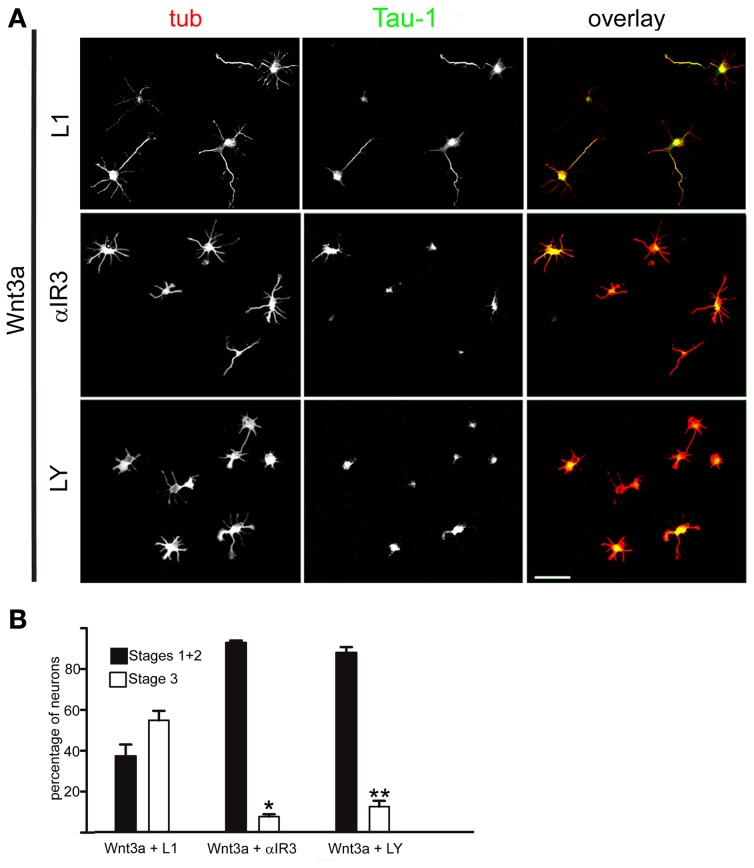
**An IGF-1r blocking antibody or the PI3k inhibitor LY294002 hindered the polarizing effects of Wnt3a. (A)** Double immunofluorescence micrographs of hippocampal neurons after 20 h in culture showing the distribution of the neuronal marker β-III tubulin (tub) and the axonal marker Tau-1, Cells were cultured in medium containing 1.35 nM Wnt3a plus an antibody to L1 (top), 1.35 nM Wnt3a plus the IGF-1r blocking antibody αIR3 (middle), or 1.35 nM Wnt3a plus the PI3k inhibitor LY294002 (bottom). **(B)** Percentage (±sem) of neurons at different stages of differentiation grown for 20 h in medium containing 1.35 nM Wnt3a plus the anti-L1 antibody, 1.35 nM Wnt3a plus the IGF-1r blocking antibody αIR3, or 1.35 nM Wnt3a plus the PI3k inhibitor LY294002 (*n* = 3 independent experiments). At least 100 cells were scored for each condition. ^*^p < 0.001 compared with Wnt3a + L1; ^**^p < 0.001 compared with Wnt3a (Figure 1B, right). Scale bar: 100 μm.

**Figure 4:**
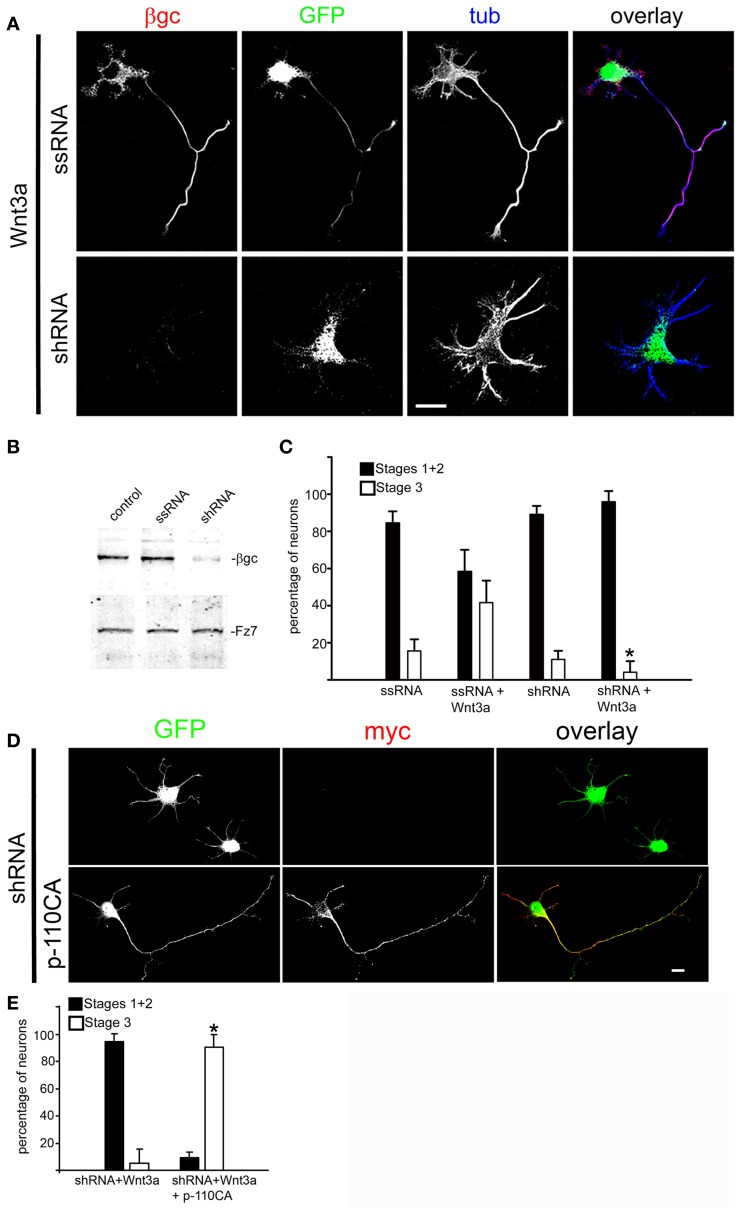
**Wnt3a polarizing effects required normal expression of IGF-1r. (A)** Triple immunofluorescence micrographs of hippocampal neurons after 24 h in culture showing the distribution of the IGF-1r β gc subunit, a scrambled sequence RNA (GFP, top) or IGF-1r targeted shRNA (GFP, bottom), and the neuronal marker β-III tubulin (tub). Cells were cultured in medium containing 1.35 nM Wnt3a. The ssRNA transfected cells (top) showed a normal morphology. In contrast, the shRNA transfected cells (bottom) did not develop an axon. **(B)** Western blots showing protein levels of β gc, the Wnt receptor Fz7 (to control specificity, also serves as loading control) and cells transfected with a scrambled RNA sequence (ssRNA), and cells transfected with IGF-1r shRNA (shRNA). We observed a substantial decrease in β gc expression in the shRNA treated cells. **(C)** Percentage (±sem) of neurons at different specific stages of differentiation grown for 20 h in culture in control medium or in the presence of 1.35 nM Wnt3a and transfected with a scrambled sequence RNA (ssRNA) or IGF-1r targeted shRNA (shRNA) (*n* = 3 independent experiments). At least 100 transfected cells (GFP positive) were scored for each condition. ^*^p < 0.005 compared to ssRNA + Wnt3a. Scale bar: 20 μm. **(D)** Immunofluorescence of hippocampal neurons after 24 h of DIV showing the distribution of myc (marker of transfection with cDNA encoding for a constitutively active form of the PI3k catalytic subunit p110). GFP is a marker of transfection efficiency with the shRNA directed to IGF-1r.Calibration bar = 20 μm. **(E)** Percentage (±sem) of neurons at different specific stages of differentiation grown for 20 h in culture in the presence of 1.35 nM Wnt3a and transfected with IGF-1r shRNA (shRNA) or co-transfected with shRNA and a constitutively active form of the PI3k catalytic subunit p110 (p110CA). *n* = 3 independent experiments. Thirty or more transfected or co-transfected cells were scored in each experiment. ^*^p < 0.02 compared to shRNA.

To further investigate the relation between Wnt3a and the IGF-1r/PI3k pathway on the establishment of neuronal polarity, we designed experiments to study the possible mechanism(s) by which Wnt3a stimulates IGF-1r and PI3k. Our results indicate that the addition of an IGF-1r blocking antibody prevented polarization of Wnt3a-challenged neurons (Figure 3A, middle), compared with cells cultured in the presence of a monoclonal antibody to the neural adhesion molecule L1 added at the same protein concentration (Figure 3A, top); therefore, most of these neurons remained at stage 2 after 20 h in culture (Figure 3B). Moreover, in loss of function experiments using a shRNA derived from the IGF-1r sequence (which uniformly silenced the expression of β gc-containing IGF-1r, shown in Figures 4A,B) (Sosa et al., 2006) we observed that the silenced cells challenged with Wnt3a failed to form axons and generated only short, minor processes (Figure 4A). To analyze this observation quantitatively, we scored the differentiation stages of neurons transfected with a scrambled sequence RNA (ssRNA) or with IGF-1r targeted shRNA after 24 h in culture. Quantification showed that only about 6% of shRNA-transfected neurons formed a discernible axon when challenged with Wnt3a, whereas 45% of controls containing ssRNA formed axons (Figure 4C). Since PI3k operates downstream of the IGF-1r and to eliminate possible artifacts due to toxic or deleterious effects of transfection with the shRNA, we cotransfected cells with the IGF-1r shRNA and with a cDNA encoding a myc-tagged constitutively active form of PI3k, p110. The results showed that co-transfection completely rescued the phenotype (Figure 4D), with over 90% of co-transfected cells exhibiting long, axon-like processes compared to less than 10% of cells transfected with IGF-1r shRNA alone (Figure 4E).

Most cells challenged with Wnt3a in the presence of the PI3k inhibitor LY294002 also failed to form axons (Figure 3A, bottom). As shown in Figure 3B, less that 10% of the neurons were in stage 3 after 24 h in culture, which was in contrast to Wnt3a-challenged neurons cultured in the absence of the PI3k inhibitor.

### Wnt3a activated the IGF-1r-PI3k pathway through the formation of an active IGF-1r-Fz7-wnt3a complex

**Figure 5:**
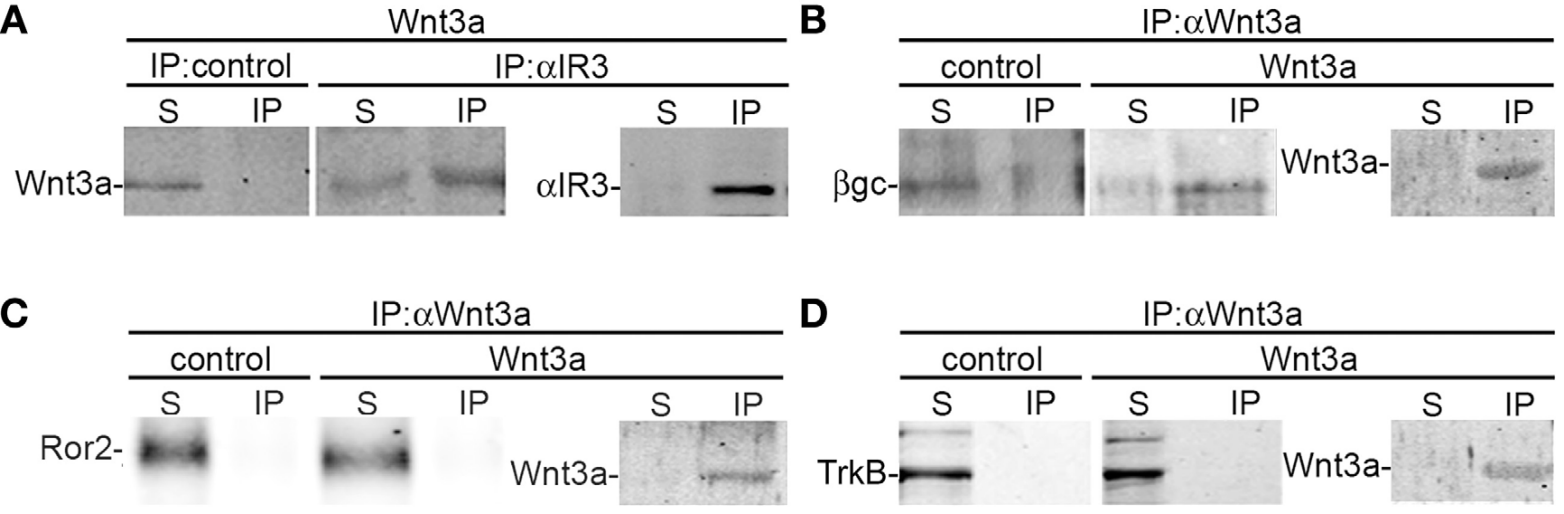
**Wnt3a interaction with IGF-1r. (A)** Growth cone particles were stimulated with Wnt3a and immunoprecipitated with a non-relevant primary antibody (IP control) or the αIR3 antibody to pull down IGF-1r. Blots were probed with an anti-Wnt3a antibody. Immunoprecipitation of αIR3 is shown to confirm successful IP and as a loading control. **(B–D)** Growth cone particles were incubated in control buffer or in the presence of 1.35 nM Wnt3a, immunoprecipitated with anti-Wnt3a and probed with **(B)** β gc antibody to reveal IGF-1r; **(C)** anti-Ror2; and **(D)** anti-TrkB (BDNF receptor prominent at the growth cone of developing neurons) Immunoprecipitation of Wnt3a is shown to confirm successful IP and as a loading control. IP, immunoprecipitate; S, soluble fraction after immunoprecipitation. All blots are representative of at least three independent experiments.

**Figure 6:**
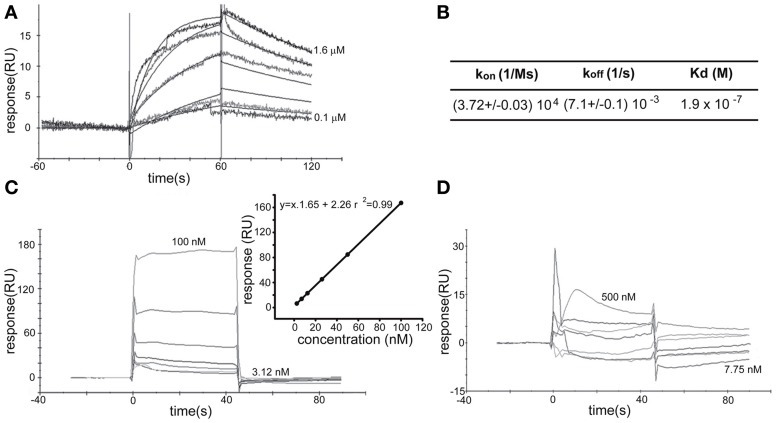
**Surface Plasmon Resonance Analysis. (A)** SPR sensogram of the interaction between hIGF-1 (1600–100 nM) and IGF-1r (1600 RU immobilized) after correction for non-specific binding. **(B)** The apparent KD for the binding of hIGF-1 to immobilized IGF-1r was 1.3 × 10^−7^ M by kinetic analysis. kon and koff from where KD was calculated are also shown. **(C)** SPR sensogram of the interaction between soluble Wnt3a (3125–100 nM) and IGF-1r (1600 RU). (Inset) The linear dependence of the response in function of the concentration of soluble Wnt3a injected over immobilized IGF-1r support the idea of non-specific direct binding. **(D)** Soluble IGF-1r (7.75–500 nM) showed no specific biding to immobilized Wnt3a.

**Figure 7:**
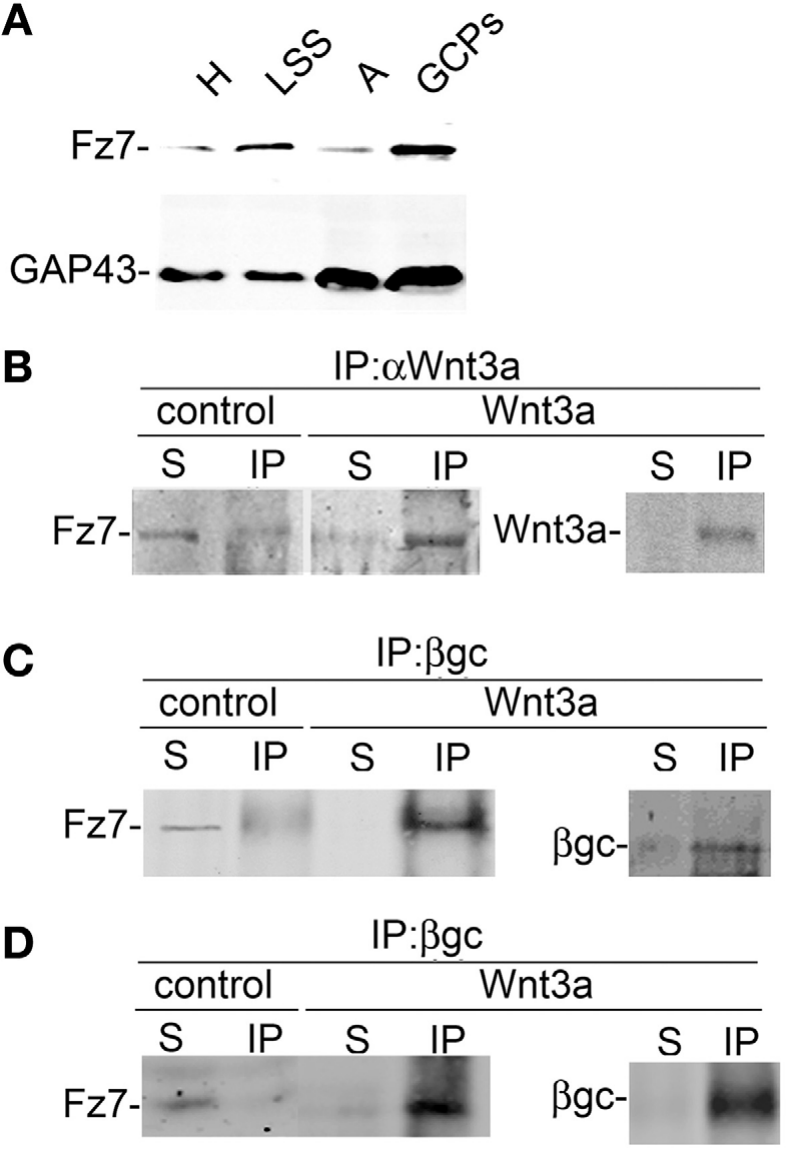
**Activation with Wnt3a triggers the formation of a IFG-1r/Wnt3a/Fz7 complex. (A)** Fz7 is enriched in GCPs. Western blot analysis of the distribution of the Wnt receptor Fz7 in different subcellular fractions of fetal brain: homogenate (H), low-speed supernatant (LSS), A-fraction (A), and growth cone particles (GCPs). Equal amounts of protein were loaded in all lines. Note the enrichment of Fz7 in GCPs around fourfold compared to (H). A blot showing the distribution of GAP-43, a protein highly enriched in GCPs, was included for comparison. **(B**,**C)** Wnt3a triggered the formation of a complex containing IGF-1r-Wnt3a-Fz7. Growth cone particles were incubated in control buffer or in the presence of 1.35 nM Wnt3a and immunoprecipitated with **(B)** Anti Wnt3a and probed with an antibody to the Wnt receptor Fz7 and **(C)** immunoprecipitated with β gc antibody to pull down IGF-1r and probed with anti Fz7. Immunoprecipitation of Wnt3a and β gc are shown to confirm successful IP and as a loading control. IP, immunoprecipitate; S, soluble fraction after immunoprecipitation. **(D)** Total protein from hippocampal neurons cultured for 20 h in the presence of 1.35 nm Wnt3a, deprived of any growth factor for 4 h, and stimulated (or not) with 1.35 nM Wnt3a for 5 min immunoprecipitated with anti β gc antibody to pull down the IGF-1r and probed with anti Fz7. Immunoprecipitation of β gc is shown to confirm successful IP and as a loading control. IP, immunoprecipitate; S, soluble fraction after immunoprecipitation. All blots are representative of at least three independent experiments.

**Figure 8:**
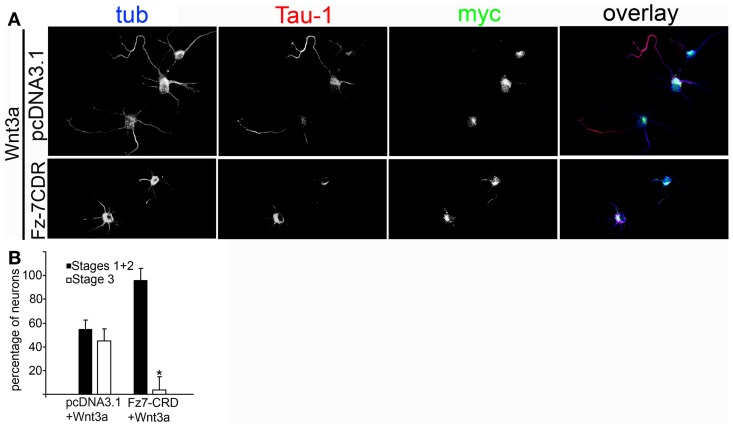
**(A)** Wnt3a polarizing effect depends on the activation of Fz7. Double immunofluorescence micrographs of hippocampal neurons incubated for 24 h in the presence of 1.35 nM Wnt3a showing the distribution of the axonal marker Tau-1 and the neuronal marker β-III-tubulin. myc is a marker of transfection with the negative dominant form of Fz7-CRD. Scale bar: 20 mm. **(B)** Percentage (±sem) of neurons at different stages of differentiation grown for 20 h in medium containing 1.35 nM Wnt3a and transfected with pcDNA3.1-myc or Fz7-CRD (*n* = 3 independent experiments). At least 20 transfected cells were scored for each condition. ^*^p < 0.01 compared with pcDNA3.1-myc.

To further study the association between Wnt3a and the IGF-1r, we performed immunoprecipitation experiments using GCPs challenged with Wnt3a and crosslinked with 3,3′-dithio*bis* (sulfosuccinimidylpropionate). The results revealed that an antibody to IGF-1r (clone αIR3) co-immunoprecipitated Wnt3a (Figure 5A). In addition, an antibody to Wnt3a co-immunoprecipitated IGF-1r in GCPs challenged with Wnt3a prior to crosslinking and immunoprecipitation (Figure 5B). Ror2 is a tyrosine kinase Wnt3a co-receptor found in non-neuronal cells (Li et al., 2008), and shown to be highly enriched at the leading edge of hippocampal pyramidal neurons in culture (Paganoni and Ferreira, 2005). After crosslinking of GCPs challenged with 1.35 nM Wnt3a, we did not observe co-immunoprecipitation of Ror2 with the Wnt3a antibody (Figure 5C). Another prominent tyrosine kinase receptor of the growth cone in developing neurons is TrkB, the BDNF receptor, which also failed to co-immunoprecipitate with the Wnt3a antibody (Figure 5D). Taken together, these results indicate that only IGF-1r co-immunoprecipitated with Wnt3a, whereas related tyrosine kinase receptors, such as Ror2 and TrkB, did not. These experiments, together with those indicating that the IGF-1r blocking antibody prevents Wnt3a axogenic effects, suggested a possible direct interaction between Wnt3a and the IGF-1r. To assess this issue we performed surface plasmon resonance (SPR) experiments. To validate the method employed, the affinity between IGF-1 and its cognate receptor, the IGF-1r, was determined. Twofold dilutions of IGF-1 (1.6–0.1 μM) were passed on immobilized IGF-1r for 60 s and response units (RU) were recorded. Dissociation was carried out using PBS. The sensogram showed specific interaction (Figure 6A) and the kinetic parameters were calculated with the Biacore T100 Evaluation software fitting to a 1:1 binding model (Figure 6B). These results are in line with those found in the literature (Surinya et al., 2008). In contrast, when different concentrations of soluble Wnt3a were passed on IGF-1r, no specific binding could be detected (Figure 6C). This could also be appreciated by the linear response in the graphic of the RU in function of the concentration (Figure 6C, inset). To preclude any possibility of inactivation of the Wnt3a binding site by immobilization of IGF-1r, an interaction analysis in the reverse orientation was made. Thus, Wnt3a was immobilized (3500 RU) and different concentration of IGF-1r were assayed as analyte. Figure 6D shows no binding of these molecules corroborating the previous result that there is no direct interaction between these proteins in an isolated system. These results raised the possibility that binding of Wnt3a to IGF-1r and its consequent activation could be achieved through the formation of a complex via binding of Wnt3a to a cognate receptor protein. We found that the Wnt family receptor Fz7 is highly enriched at the growth cone of differentiating neurons (Figure 7A). As expected, the Wnt3a antibody co-immunoprecipitated Fz7 (Figure 7B). Moreover, an antibody to the β gc subunit of IGF-1r co-immunoprecipitated Fz7 only when the GCPs were preincubated with Wnt3a (Figure 7C). In addition, we obtained similar results by immunoprecipitating proteins from cultures of hippocampal neurons deprived of growth factors for 4 h and kept in control conditions (no co-immunoprecipitation was observed) or stimulated for 5 min with Wnt3a (Figure 7D). It follows that binding of Wnt3a to Fz7 promoted the formation of an IGF-1r (active)-Wnt3a-Fz7 complex at the growth cone of hippocampal pyramidal neurons in culture. To get more information about the characteristics of this complex we performed SPR experiments passing the extracellular moiety of Fz7 (aa 45–169) preincubated (or not) 1:1 with Wnt3a on immobilized IGF-1r (extracellular moiety). No specific interaction between the two receptors was detected in this experiment (not shown). Finally, we performed experiments in order to obtain direct evidence about the participation of the Fz7 receptor in Wnt3a induced neuronal polarization. For that purpose, we transfected hippocampal pyramidal neurons with the myc-tagged dominant negative Fz7 CRD which uncouples the binding of Wnts to Fz7 from the receptor activation (Wei et al., 2011). The results of these experiments are shown in Figure 8, a quantification of the transfected neurons indicated that less than 5% of the cells transfected with Fz7 CRD reached stage 3 of differentiation, exhibiting a Tau-1 positive axon, compared to over 60% of the control cells transfected with the plasmid expressing only myc (Figure 8B).

## Discussion

Secreted Wnts interact with their membrane receptors and activate three different intracellular cascades: (i) The canonical or Wnt/β-catenin pathway, which involves cytosolic stabilization of β-catenin, its translocation to the nucleus, and the transcriptional activation of specific genes; (ii) The planar cell polarity pathway, which involves the activation of small Rho-GTPases and kinases that modulate cell orientation and organization; and (iii) The Wnt/calcium pathway, which regulates intracellular levels of calcium and the activation of Ca^+2-sensitive^ kinases (Ciani and Salinas, 2005). To activate intracellular pathways, Wnts interact with different families of receptors, such as seven-span transmembrane receptor molecules known as Frizzled, the co-receptors from the family of low-density lipoprotein receptor-related proteins (LRP 5/6), and two atypical tyrosine-kinase receptors Ror1 or 2 and Ryk (Angers and Moon, 2009). Wnts signal through the novel Ryk receptor via multiple mechanisms, including nuclear translocation of their intracellular domains and activation of pathways employing Src family kinases and members of the canonical Wnt pathway (Fradkin et al., 2010). It has also been shown that one member of the Wnt family involved in hippocampal formation, Wnt3a, induces Akt activation via direct activation of PI3k in fibroblasts (Kim et al., 2007). In addition, Wnt3a has been shown to activate Akt in PC12 cells (Fukumoto et al., 2001). The receptor system(s) involved in PI3k activation by Wnt3a, however, have not been identified. We have previously shown that activation of IGF-1r (and activation of PI3k) is essential for polarization of cultured hippocampal pyramidal neurons (Sosa et al., 2006). The results shown here demonstrate that Wnt3a can promote initial axonal outgrowth and neuronal polarization in the absence of IGF-1 and also suggest that Wnt3a is important for axon maintenance and further axonal outgrowth after polarization. These results and previously published data prompted us to investigate the possible activation of PI3k by Wnt3a in cultured hippocampal neurons to identify the receptor(s) involved in PI3k activation, and determine Wnt3a consequences on initial axonal outgrowth and neuronal polarization. Surprisingly, we found that Wnt3a could trigger a robust and significant activation of IGF-1r, PI3k, and Akt in GCPs (in the absence of IGF-1 or high insulin; Figures 2A–C). Activation of IGF-1r and PI3k was polarized to one neurite of cells in stage 2 of differentiation that had not exhibited a noticeable axon (Figure 2C). Moreover, challenge with Wnt3a in defined medium that did not contain any growth factors except insulin (5 nM) was sufficient to trigger the establishment of neuronal polarity (Figure 1). Wnt3a polarizing activity required the presence of activatable, membrane inserted IGF-1r (Figures 3, 4). Crosslinking and immunoprecipitation experiments of GCPs challenged with Wnt3a suggested interaction of Wnt3a with IGF-1r. However, by SPR experiments no direct interaction between IGF-1r and Wnt3a was observed neither when IGF-1r was immobilized nor when Wnt3a was attached to the chip surface (Figures 6C,D). Thus, SPR results suggested no direct, specific interaction between these proteins in an isolated system. Finally, by immunoprecipitation experiments we observed that stimulation with Wnt3a triggered the formation of a complex containing IGF-1r-Wnt3a-Fz7. Moreover, we demonstrated that Fz7 is essential for the Wnt3a observed effects in hippocampal neurons. It follows that axogenic effects of Wnt3a are due to a polarized cross-activation of the IGF-1r/PI3k pathway. Interestingly, there is a significant temporal overlap between high Wnt3a expression (Lee et al., 2000) and the initial outgrowth of axons in the hippocampus “*in situ*” (Fletcher and Banker, 1989). Since the expression of IGF-1 gene and its transcript is high in the developing brain but decreases significantly in the adult (Rotwein et al., 1988; Leroith et al., 1993), the activation of the IGF-1r by other growths factor, such as Wnts, could significantly expand the time window for the activation of this receptor. A cross-talk between the insulin receptor (homologous to the IGF-1r) and Wnt have been recently shown in non-neuronal cells involving co-receptor low density lipoprotein receptor-related protein-5 (Palsgaard et al., 2012). Besides its central role in the establishment of neuronal polarity, the PI3k-Akt pathways is involved in the regulation of many different phenomena in the central nervous system, including cell division (during neuronal proliferation), large scale cellular remodeling (during differentiation-including dendrite and synapses development), and synaptic specific changes during plasticity-(Knafo and Esteban, 2012). Many neurobehavioral disorders arise as a consequence of subtle developmental abnormalities and aberrant PI3k signaling has been indicated by many studies to be a contributing factor to the pathophysiology of disorders such as schizophrenia and autism (Waite and Eickholt, 2010). Moreover, PI3k signaling strongly enhances the resistance of several neuron types against aggressions such us oxidative damage and hypoxia. It follows that activation of PI3k by Wnt3a, in neurons, can have a significant physiological and/or pathophysiological impact.

As discussed above, numerous receptors and co-receptors have been identified for the members of the Wnt family growth factors, and Frizzled-3 (Fz3) has been proposed to have a role in neurite outgrowth and nervous differentiation of glioblastoma cells (Rampazzo et al., 2013). Also, a mouse Fz3 knock out exhibited defects in axonal outgrowth and guidance (Wang et al., 2006). In our experimental system, however, we did not find detectable expression of Fz3 using two different antibodies (not shown) at least inside the times of culture used in this study. In contrast, we found high levels of Fz7 in neurons dissociated from hippocampi at embryonic day 18 (E18) cultured for 20 h and GCPs prepared from fetal rat brain (E18). A high level of expression of Fz7 was also found in rat hippocampi (E18) (Varela-Nallar et al., 2012).

As mentioned above, we did not find evidences indicating a direct interaction of the IGF-1r with Fz7 bound to Wnt3a using SPR. Several reasons can explain this observation, including: (i) That the presence of transmembrane and/or intracellular regions of one or both receptors are necessary for the interaction, since we used the extracellular moiety of the receptors in our SPR experiments; (ii) That the interaction between the receptors is not direct. There are published results that can support this possibility. For example, cross activation of the LRP6 receptor by Wnts depends on the formation of a complex containing Frizzled, LRP6, Disheveled-1, and the axin Gsk3 complex (Zeng et al., 2008). Hence, more investigation will be needed to obtain a complete picture of the components of the IGF-1r-Wnt3a-Fz7 complex and the characteristics of the binding of these different components. Interestingly, it has been recently published that IGF-1 signaling interacts with canonical Wnt signaling to promote neuronal proliferation (Hu et al., 2012).

**Figure 9:**
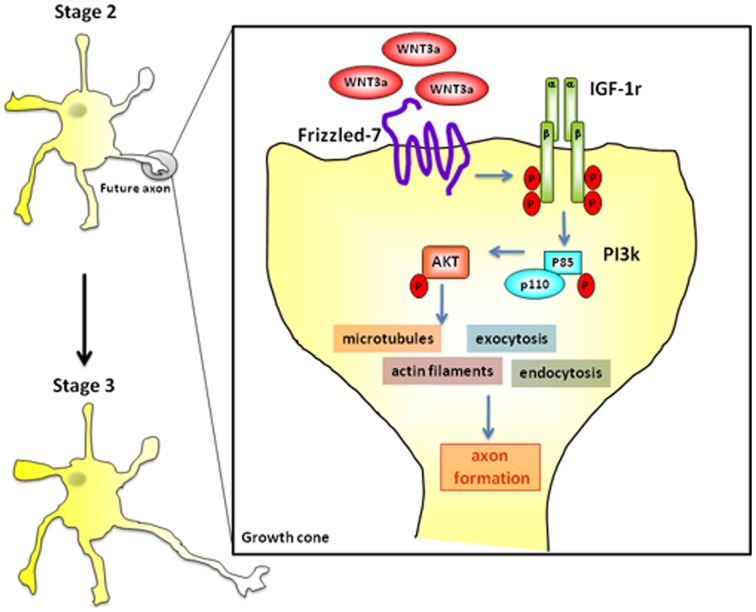
**Wnt3a binds to Fz7 and cross-activate the IGF-1-PI3k-Akt pathway inducing initial axonal outgrowth and neuronal polarization.** Challenging with 1.35 nM Wnt3a resulted in a polarized distribution of active (phosphorylated) IGF-1r and phosphorylated p85, the PI3k regulatory subunit, to one neurite of cells in stage 2 (see Figure 2F).

Taken together, our results indicate that cross activation of the IGF-1r/PI3k pathway by Wnt3a is sufficient for the establishment of neuronal polarity by regulating initial axonal elongation (a cartoon showing the proposed activation pathway is shown in Figure 9). Indeed, the present results suggest that Wnt3a, together with IGF-1, is one of the growth factors initiating axonal outgrowth in hippocampal neurons.

## Author contributions

Conceived and designed the experiments: María E. Bernis, Mariana Oksdath, Sebastián Dupraz, Alvaro Nieto Guil, Marisa M. Fernández, Emilio L. Malchiodi, Silvana B. Rosso, and Santiago Quiroga. Performed the experiments: María E. Bernis, Mariana Oksdath, Sebastián Dupraz, Alvaro Nieto Guil, and Marisa M. Fernández. Analyzed the data: María E. Bernis, Mariana Oksdath, Sebastián Dupraz, Alvaro Nieto Guil, Marisa M. Fernández, Emilio L. Malchiodi, Silvana B. Rosso, and Santiago Quiroga. Contributed reagents/materials/analysis tools: Sebastián Dupraz, Silvana B. Rosso, Marisa M. Fernández, and Emilio L. Malchiodi. Wrote the paper: María E. Bernis, Sebastián Dupraz, Marisa M. Fernández, Emilio L. Malchiodi, Silvana B. Rosso, and Santiago Quiroga.

### Conflict of interest statement

The authors declare that the research was conducted in the absence of any commercial or financial relationships that could be construed as a potential conflict of interest.
